# Revealing the Changes in Saliva and Serum Proteins of Pigs with Meningitis Caused by Streptococcus Suis: A Proteomic Approach

**DOI:** 10.3390/ijms232213700

**Published:** 2022-11-08

**Authors:** María José López-Martínez, Anđelo Beletić, Josipa Kuleš, Dina Rešetar-Maslov, Ivana Rubić, Vladimir Mrljak, Edgar Garcia Manzanilla, Elena Goyena, Silvia Martínez-Subiela, José Joaquín Cerón, Alberto Muñoz-Prieto

**Affiliations:** 1 Interdisciplinary Laboratory of Clinical Analysis of the University of Murcia (INTERLAB-UMU), Department of Animal Medicine and Surgery, Veterinary School, Regional Campus of International Excellence Mare Nostrum, University of Murcia, 30100 Murcia, Spain; 2 Clinic for Internal Diseases, Faculty of Veterinary Medicine, University of Zagreb, Heinzelova 55, 10000 Zagreb, Croatia; 3 Department of Chemistry and Biochemistry, Faculty of Veterinary Medicine, University of Zagreb, Heinzelova 55, 10000 Zagreb, Croatia; 4 Pig Development Department, The Irish Food and Agriculture Authority, Teagasc, Moorepark, P61 C996 Fermoy, Ireland; 5 School of Veterinary Medicine, University College Dublin, Belfield, D04 W6F6 Dublin, Ireland; 6 Department of Animal Health, Faculty of Veterinary Medicine, University of Murcia, 30100 Murcia, Spain

**Keywords:** *Streptococcus suis*, meningitis, pigs, saliva, proteomics, biomarkers

## Abstract

Meningitis due to *Streptococcus suis* causes high mortality and morbidity on pig farms and has increasing zoonotic potential worldwide. Saliva proteome analysis would potentially be useful in elucidating pathophysiological changes and mining for new biomarkers to diagnose and monitor *S. suis* infection. The objective of this study was to investigate the changes in the salivary and serum proteome profile of piglets with meningitis. The LC-MS/MS TMT proteomic approach was used to analyze saliva and serum samples from 20 male piglets: 10 with meningitis and 10 healthy. In saliva, 11 proteins had higher and 10 had lower relative abundance in piglets with meningitis. The proteins with the highest relative abundance were metavinculin (VCL) and desmocollin-2 (DSC2). Adenosine deaminase (ADA) was selected for validation using a spectrophotometric assay and demonstrated excellent performance in the differentiation between healthy and pigs with meningitis due to *S. suis*. In serum, the most protruding changes occurred for one SERPIN and haptoglobin (HP). In saliva and serum, the highest number of proteins with altered abundance were linked, via the enrichment analysis, with platelet and neutrophil pathways. Overall, meningitis caused by *S. suis* resulted in specific proteome changes in saliva and serum, reflecting different pathophysiological mechanisms, and marking new potential biomarkers for this infection.

## 1. Introduction

The *Streptococcus suis* is a Gram-positive bacteria considered one of the most important swine pathogens. It is associated with significant mortality and morbidity on pig farms, with an average of 14% post-weaning mortality in a previous study in Canada [1], being responsible for major economic losses in the swine industry [2]. In addition to meningitis, which is the common clinical manifestation, *S. suis* infection also can cause arthritis, pneumonia, or endocarditis [3]. It also has significant zoonotic potential, and infection in humans has similar clinical features as in pigs [4]. The number of cases in humans is increasing worldwide with cases of *S. suis* infections reported in more than 30 countries or regions of the world, mainly in Southeast Asia [5]. The rising incidence of human infections is associated with the increasing number of pig farms and the lack of preventive measures to avoid the infection, such as consuming uncooked pig products or slaughtering practices without satisfactory preventive barriers [6].

Proteomics represents a comprehensive combination of analytical approaches endeavoring to decipher qualitative and quantitative protein composition in a sample [7]. Protein changes in the serum of piglets with an experimentally induced infection with *S. suis* were studied through a proteomic approach [8]. In this report, the authors highlighted ten serum proteins as potential biomarkers due to the different levels in the meningitis group compared with the control group. ADP ribosylation factor 4, immunoglobulin lambda-like polypeptide 5, phosphoglycerate mutase 1, and thioredoxin-1 were among the nine proteins with higher levels in the meningitis group, while complement component 4 binding protein alpha was the only protein with a lower level in piglets with the disease.

Saliva is a fluid attracting growing attention in the research of biomarkers for health and welfare evaluation. The easy sampling procedure, with minimal stress and no need for complex equipment, makes it convenient for large-scale sampling [9]. Proteomic studies using saliva have been published for the selected diseases in various species such as equine gastric ulcer syndrome in horses [10] or mastitis in cows [11] to gain knowledge about the pathophysiology of diseases and to find potential biomarkers. In pigs, a recent proteomic study explored the changes in saliva that occurs in sepsis induced by LPS administration [12]. In these studies, proteomic methodology relied on liquid chromatography-tandem mass spectrometry (LC-MS/MS) analysis of peptides labelled with isobaric tandem mass tags (TMT), which allowed an accurate simultaneous relative quantification of multiple proteins [13].

However, to the best of our knowledge, no proteomic studies exploring the changes of protein content in the saliva of pigs with meningitis caused by *S. suis* have been performed. Thus, the main objective of this study was to investigate if the infection by *S. suis* produces changes in the salivary protein composition of piglets. Additionally, the serum proteomic profile was analyzed to compare with those changes observed in saliva.

## 2. Results

### 2.1. Salivary Proteomic Profile in Piglets with Meningitis

**Figure 1:**
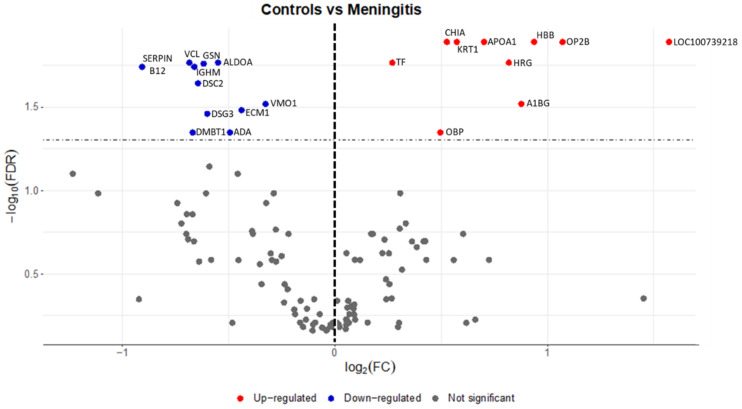
Volcano plot showing salivary proteins with the higher (red color) and lower (blue color) relative abundance between controls and pigs with meningitis. Gene symbols for the proteins are given on the right. Abbreviations: A1BG—Alpha-1B-glycoprotein, ADA—Adenosine aminohydrolase, ALDOA—Fructose-bisphosphate aldolase, APOA1—Apolipoprotein A-I, CHIA—Chitinase, DMBT1—Isoform 2 of Deleted in malignant brain tumors 1 protein, DSC2—Desmocollin 2, DSG3—Desmoglein 3, ECM1—Extracellular matrix protein 1, GSN—Actin-depolymerizing factor, HBB—Hemoglobin subunit beta, HRG—Cystatin domain-containing protein, IGHM—Immunoglobulin heavy constant mu, KRT1—Cytokeratin-1, LOC100739218—Double-headed protease inhibitor, submandibular gland-like, OBP—Odorant-binding protein, OBP2B—Lipocln_cytosolic_FA-bd_domdomain-containing protein, SERPINB12—SERPIN domain-containing protein, TF—Serotransferrin, VCL—Metavinculin, VMO1—Vitelline membrane outer layer protein 1.

**Figure 2:**
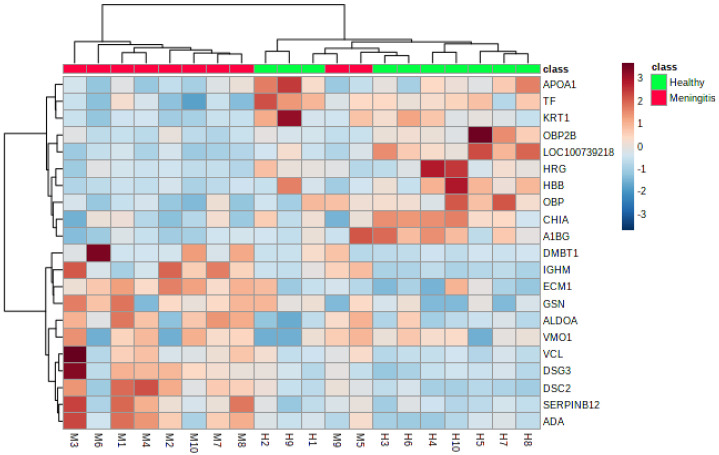
Hierarchical cluster analysis based on the proteins with the different relative abundance in saliva of the piglets with meningitis (red board) and the healthy piglets (green board). The red color represents the increased relative abundance, and the blue corresponds to the decreased relative abundance in meningitis versus the healthy group. Abbreviations: A1BG—Alpha-1B-glycoprotein, ADA—Adenosine aminohydrolase, ALDOA—Fructose-bisphosphate aldolase, APOA1—Apolipoprotein A-I, CHIA—Chitinase, DMBT1—Isoform 2 of Deleted in malignant brain tumors 1 protein, DSC2—Desmocollin 2, DSG3—Desmoglein 3, ECM1—Extracellular matrix protein 1, GSN—Actin-depolymerizing factor, HBB—Hemoglobin subunit beta, HRG—Cystatin domain-containing protein, IGHM—Immunoglobulin heavy constant mu, KRT1—Cytokeratin-1, LOC100739218—Double-headed protease inhibitor, submandibular gland-like, OBP—Odorant-binding protein, OBP2B—Lipocln_cytosolic_FA-bd_domdomain-containing protein, SERPINB12—SERPIN domain-containing protein, TF—Serotransferrin, VCL—Metavinculin, VMO1—Vitelline membrane outer layer protein 1.

A total of 21 proteins showed different relative abundances between control and disease groups (Supplementary Table S1, Figure 1 and Figure 2). The highest fold changes (FC) were: metavinculin (VCL), desmocollin 2 (DSC2), immunoglobulin heavy constant mu (IGHM), fructose-biphosphate aldolase (ALDOA) and a serpin domain-containing protein (SERPINB12). In the case of salivary proteins with lower relative abundance in MP, those with the most evident FC were lipocalin cytosolic FA-bd domain-containing protein (OBP2B), hemoglobin subunit beta (HBB), and double-headed protease inhibitor, submandibular gland-like (LOC100739218).

**Figure 3:**
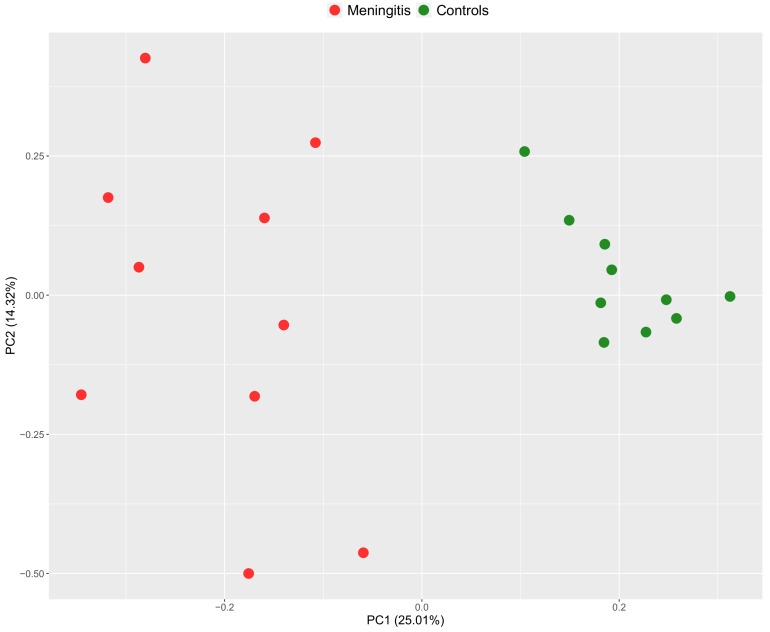
Principal component analysis (PCA) score plot of saliva samples showing the distribution of groups.

In addition, the PCA plot clearly indicated that samples formed separated clusters between groups (Figure 3).

The differentially expressed proteins in saliva between meningitis and healthy groups were used for analysis in terms of functional clusters, according to the PANTHER classification system (http://www.pantherdb.org, accessed on 20 July 2022) (Supplementary Table S2). The identified differentially abundant proteins had four molecular functions, with binding (GO:0005488) and catalytic activity (GO:0003824) as the most representative. Twelve biological processes were associated with these proteins, whereby the cellular process (GO:0009987) and metabolic process (GO:0008152) were those with the highest percentage of genes associated.

The pathway enrichment analysis showed alterations in nine different pathways, such as platelet degranulation, response to elevated platelet cytosolic Ca2+, platelet activation, signaling and aggregation, neutrophil degranulation, and innate immunity pathways (Table 1).

### 2.2. Serum Proteomic Profile in Piglets with Meningitis

**Figure 4:**
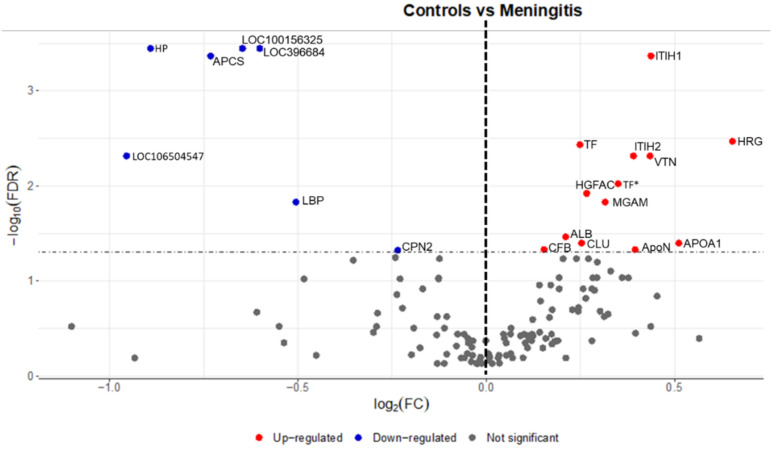
Volcano plot showing serum proteins with the higher (red color) and lower (blue color) relative abundance between controls and pigs with meningitis. Gene symbols for the proteins are given on the right. Abbreviations: *—identified after blasting in UniProt, ALB—Albumin (Fragment), APCS—Serum amyloid P-component, APOA1—Apolipoprotein A-I, ApoN—Ovarian and testicular apolipoprotein N, CFB—C3/C5 convertase, CLU—Clusterin, CPN2—Carboxypeptidase N subunit 2, HGFAC—Hepatocyte growth factor activator isoform 2 preproprotein, HP—Haptoglobin, HRG—Histidine-rich glycoprotein, ITIH1—Inter-alpha-trypsin inhibitor heavy chain H1 isoform, ITIH2—Inter-alpha-trypsin inhibitor heavy chain H2, LBP—Lipopolysaccharide-binding protein, LOC100156325—SERPIN domain-containing protein, LOC106504547—SERPIN domain-containing protein, LOC396684—SERPIN domain-containing protein, MGAM—Maltase-glucoamylase (intestinal), TF—Serotransferrin/Beta-1 metal-binding globulin, VTN—Vitronectin.

**Figure 5:**
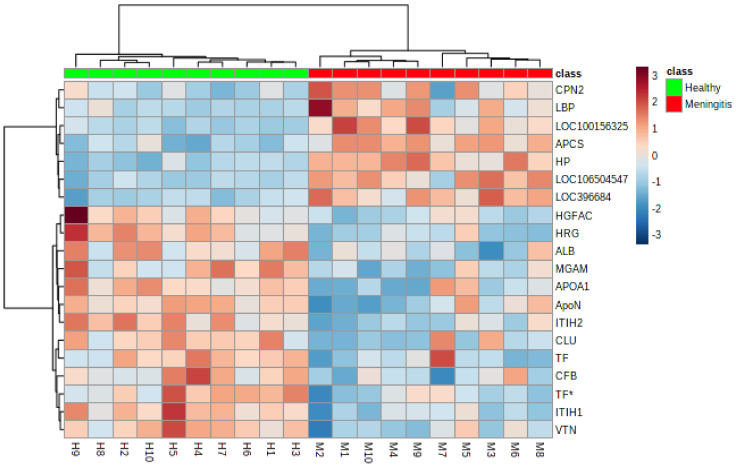
Hierarchical cluster analysis based on the proteins with the different relative abundance in serum of the piglets with meningitis (red board) and the healthy piglets (green board). The red color represents the increased relative abundance, and the blue corresponds to the decreased relative abundance in meningitis versus the healthy group. Abbreviations: *—identified after blasting in UniProt, ALB—Albumin (Fragment), APCS—Serum amyloid P-component, APOA1—Apolipoprotein A-I, ApoN—Ovarian and testicular apolipoprotein N, CFB—C3/C5 convertase, CLU—Clusterin, CPN2—Carboxypeptidase N subunit 2, HGFAC—Hepatocyte growth factor activator isoform 2 preproprotein, HP—Haptoglobin, HRG—Histidine-rich glycoprotein, ITIH1—Inter-alpha-trypsin inhibitor heavy chain H1 isoform, ITIH2—Inter-alpha-trypsin inhibitor heavy chain H2, LBP—Lipopolysaccharide-binding protein, LOC100156325—SERPIN domain-containing protein, LOC106504547—SERPIN domain-containing protein, LOC396684—SERPIN domain-containing protein, MGAM—Maltase-glucoamylase (intestinal), TF—Serotransferrin/Beta-1 metal-binding globulin, VTN—Vitronectin.

A total of 20 proteins were different in their relative abundances between the control and disease groups (Supplementary Table S3, Figure 4 and Figure 5). Proteins with the highest abundance were three serpin domain-containing proteins (LOC106504547, LOC396684*, and LOC100156325), haptoglobin (HAPT), serum amyloid P-component (APCS), and lipopolysaccharide-binding protein (LBP). Within the serum proteins with lower abundance in the MP group, the most evident FC were observed in histidine-rich glycoprotein (HRG), apolipoprotein A-I (APOA1), and inter-alpha-trypsin inhibitor heavy chain H1 isoform a preproprotein (ITIH2).

**Figure 6:**
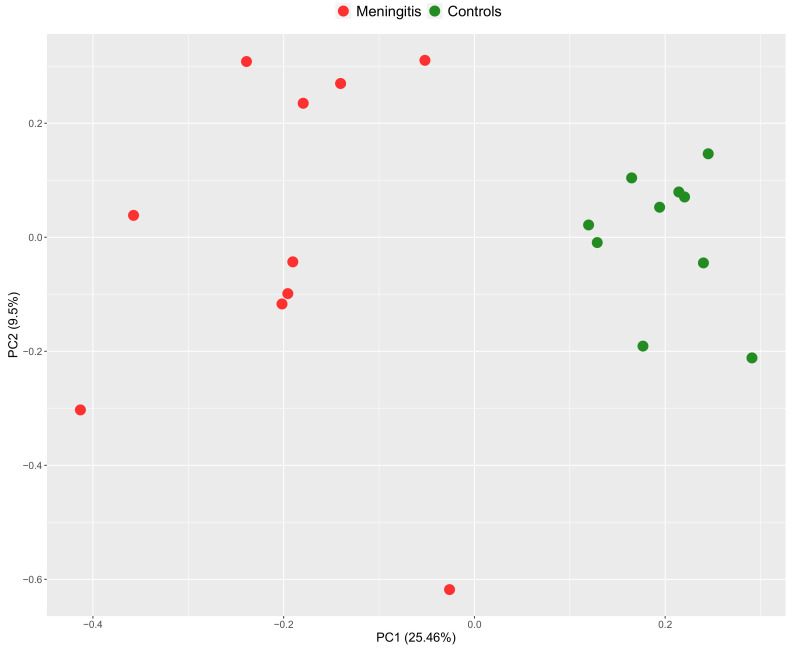
Principal component analysis (PCA) score plot of serum samples showing the distribution of groups.

The PCA plot showed no signs of overlapping in the samples of each group; therefore, two different clusters were observed between the disease and control groups (Figure 6).

The data from GO for the differentially abundant proteins in serum between pigs with meningitis and healthy pigs are reported in Supplementary Table S4. The identified differentially abundant proteins had three molecular functions, including binding (GO:0005488), catalytic activity (GO:0003824), and molecular function regulator (GO:0098772). Moreover, they participated in a total of 13 biological processes, with the cellular process (GO:0009987), response to stimulus (GO:0050896), and biological regulation (GO:0065007) reported as those with the higher percentage of genes associated.

Reactome pathway analysis of the statistically significantly different serum proteins between pigs with meningitis and healthy pigs showed alterations in 11 different pathways, whereby the most representative were those associated with platelet and neutrophil activation and complement system (Table 2).

### 2.3. Validation Study: Adenosine Deaminase (ADA) Activity of Saliva in Pigs with Meningitis

**Figure 7:**
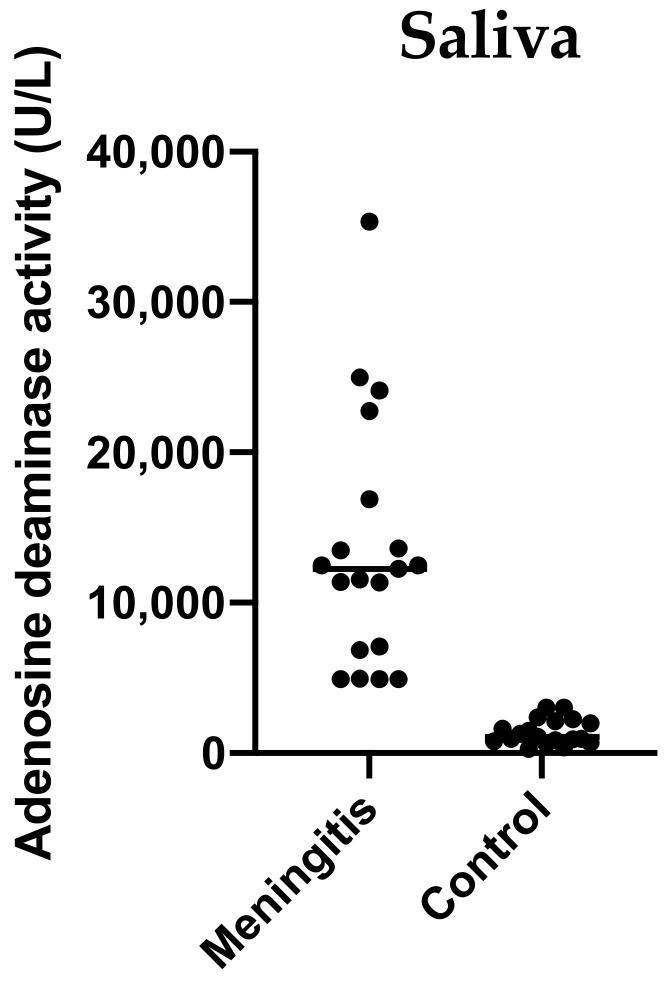
Adenosine deaminase (ADA) activity of saliva in the group of meningitis compared with control. Individual values are represented as dots and horizontal lines indicated median values.

The measurements of salivary ADA activity showed significantly higher activity levels in pigs with meningitis caused by *S. suis* (median 12,480 U/L, minimum–maximum range 4928–35,360 U/L) compared with healthy pigs (median 1072 U/L, minimum–maximum range 281.6–3008 U/L) (*p* < 0.001) (Figure 7).

The ROC analysis indicated an excellent performance of ADA salivary activity with an area under the curve (95% confidence interval (CI)) of 0.983 (0.948–1.000). The calculated cut-off of 3106 U/L distinguished groups of piglets with meningitis and healthy piglets with equal sensitivity and specificity of 95% (75–100%).

## 3. Discussion

In this study, the changes in the salivary and serum proteome of pigs with meningitis caused by *S. suis* were evaluated. The high-resolution proteomic analysis showed a total of 21 salivary and 20 serum proteins changed in abundance in MP compared with HP.

### 3.1. Changes in Salivary Proteins in Pigs with Meningitis

In the case of saliva, of these 21 proteins, 11 were increased and 10 were decreased when pigs had meningitis. The molecular functions of these proteins were mainly related to binding capacity (40.7%) and catalytic activity (25.9%), and the most upregulated were VCL and DSC2.

VCL is a cytoskeletal protein considered a part of the complex that anchors actin to the cell membrane present among other tissues in the cardiac muscle [14]. Generally, vinculins play a key role in regulating cell adhesion, motility, and muscle endurance [15]. The presence of vinculins in muscle has been related to the contractile need of the cells; thus, the greater the contractile need of the muscle is, the greater the expression of the protein will be [16]. The increased expression of VCL in saliva could potentially be associated with muscle damage and seizures that usually are presented in the case of meningitis [14]. In this line, in our study, we have also observed an upregulation in the actin-depolymerizing factor (GSN) whose family is involved in the muscle actin filament organization and muscle contraction [17].

DSC2 is a cadherin that belongs to the desmocollins family. Molecules from the cadherin family are involved in the Ca2+-dependent mechanism for cell–cell adhesion [18], and DSC2 is only present in the desmosomes. The presence of DSC2 was related to cardiac alterations being associated with myocardial inflammation and fibrotic remodeling in mice [19] or in rhythm problems in humans [19,20]. The increased DSC2 in the saliva of pigs with meningitis could indicate possible myocardial damage since it is documented that cardiac injury is associated with the meningitis process in *S. suis* infections [21].

ADA was selected to validate the proteomic results in a larger population of pigs with meningitis due to the availability of an automated spectrophotometric assay validated for pigs [22]. ADA is a biomarker for inflammatory and immune disorders in pigs [23]. The increased ADA abundances in the saliva of pigs with meningitis could be due to the immune activation produced by the *S. suis* infection. This protein did not increase in the serum, confirming the different behavior in these two fluids that was previously documented [24]. Further studies should be conducted to explore the ability of ADA to detect non-sick carriers or to predict the outcome of the disease.

Although it was not specifically validated in our study, the increase found in ALDOA in the saliva is in agreement with a recent report in which increases in ALDOA were detected in the saliva of pigs with meningitis with a spectrophotometric assay [12] and in the serum of pigs with meningitis experimentally induced by *S. suis* [8].

Among the proteins with the lowest FC in the saliva of piglets with meningitis, OBP2B and HBB might be the most relevant. OBP2B belongs to the lipocalin family that is secreted by mandibular and submandibular glands in pigs [25]. Members of the lipocalin family like neutrophil gelatinase-associated lipocalin (NGAL) exerted a protective effect on the brain during inflammatory conditions in mice [26], and its deficiency results in a high susceptibility to worsening sepsis as was postulated previously [27].

HBB is a hemoglobin subunit, and this is the major heme protein of erythrocytes, facilitating the transport of oxygen and carbon dioxide in the blood [28]. An increase in serum HBB was considered an early predictor of sepsis in humans [29], and in pigs, serum HBB level increased in sepsis induced by lipopolysaccharide (LPS) administration [30]. However, we found a decreased expression of HBB in the saliva of pigs with meningitis, and a decreased level of this protein has been reported in the saliva of other animal species, like horses with acute abdominal disease [31]. Our data could be indicating a divergence in the behavior of HBB in saliva and serum in this condition that should be further explored.

The altered GO terms showed an intense activation of platelets during meningitis which is in line with previous data indicating the implication of platelets during sepsis [32]. In fact, it was reported the inhibition of platelet activation as a potential therapy in septic patients to prevent endothelial damage and organ failure [33]. This GO term would indicate that platelets can be implied in the progress of the disease and the severity of its clinical manifestations in pigs.

As previously mentioned, some proteins that changed in pigs with meningitis, varied also in sepsis due to LPS injection, such as ALDOA, SERPINB12 or OBP2B [12]. However, in our study, there were specific proteins only showing changes in pigs with meningitis but not in pigs with sepsis due to LPS administration, like VCL, DSC2 or HBB. Further studies are needed to find proteins that could specifically and selectively change in this disease and differentiate it from other septic conditions.

### 3.2. Changes in Serum Proteins in Pigs with Meningitis

In serum, a total of 23 proteins were changed in their abundance in pigs with meningitis. In our study, the numbers of proteins with altered relative abundance were similar in saliva (21) and serum (23), but these proteins were different, with the exception of ALDOA, and serotransferrin (TF) which were downregulated in both fluids. However, the molecular function of the serum proteins that changed was associated with binding capacity (27.6%) and catalytic activity (27.6%) in a similar manner to saliva. The fact that different proteins were detected in saliva and serum may indicate that both biofluids would be providing complementary, although maybe not necessary correlative, information on the pathophysiology features of meningitis caused by *S. suis*. A similar observation occurred previously when saliva and serum were analyzed in horses with ulcers [10], cows with mastitis [11], or dogs with pyometra [34]. This highlights the hypothesis that saliva and serum could reflect different, but complementary pathophysiological features that occur in diseases [35].

Among the proteins that changed in the serum of pigs with meningitis, there were 7 upregulated and 13 downregulated. The most upregulated proteins were a serpin domain-containing protein (LOC106504547), HAPT, and APCS. Within the downregulated proteins, the most protruding changes were observed in histidine-rich glycoprotein (HRG), and apolipoprotein A-I (APOA1).

Three SERPINs different from the SERPINB12, found in saliva, had increased relative abundance in serum of pigs with meningitis (LOC106504547, LOC396684, and LOC100156325). These increases could be related to the protective role of these proteins. Two of these serpins (LOC106504547, and LOC396684) were previously reported upregulated in the serum of pigs with sepsis [12] and therefore could be associated with septic conditions.

HP is a plasma protein that tightly captures hemoglobin (Hb) during hemolysis [36]. It is considered a moderate acute phase protein in swine [37,38]. The concentration of haptoglobin has been previously described as increased in inflammatory and infectious processes such as the administration of LPS [12,30,39,40], and viral infections [41].

APCS is a glycoprotein that belongs to the family of pentraxins closely related to C-reactive protein (CRP) [42] that regulates several aspects of the innate immune system such as the inhibition of fibrocytes and neutrophil adhesion to extracellular matrix proteins or promoting the immuno-regulatory macrophages [43]. The main features of APCS lie in the modulation of the humoral innate immune system spanning the complement system, inflammation, and coagulation [44]. In line with these data are the pathway enrichment analysis results indicating APCS as one of the main proteins associated with the complement cascade pathway.

Within the downregulated proteins, the most protruding changes were observed in histidine-rich glycoprotein (HRG), and apolipoprotein A-I (APOA1).

HRG is a 75 kDa glycoprotein synthesized in the liver and released into the bloodstream to modulate sepsis-related biological reactions by binding to several substances (like heparin, factor XII, fibrinogen, thrombospondin, IgG, C1q, among others) and cells [45]. The HRG was also observed downregulated in the serum of pigs with sepsis in a previous study [12]. We also showed the implication of HRG in the platelet degranulation pathway. The decreased abundances of serum HRG may lead to a hypercoagulative state, fibrinolysis, and enhanced immune response that have been reported before in sepsis [46]. Additionally, HRG could be of particular importance in bacterial infection due to its ability to bind these pathogens [47]. Thus, the reduction of HRG might suggest a depleted capacity of the organisms to “fight” against bacterial infection and a predisposition to complications associated with it.

APOA1 is a negative acute phase protein and the major protein of high-density lipoprotein (HDL). Previous studies reported the anti-inflammatory and antithrombotic properties inherent to this protein, due to its ability to reduce the production of proinflammatory cytokines and chemokines [48]. APOA1 was also found down-regulated in the serum of pigs with sepsis induced by LPS injection [12], and its reduction in serum was documented after an experimental infection by *S. suis* [49]. APOA1 is considered a good predictor of infectious disease in pigs, due to levels dropping rapidly when the infection appears [50]. In the same manner that occurred with HRG, the relation observed with the platelet degranulation pathway may suggest that APOA1 could be a potential indicator of the presence of possible complications or increased severity in sepsis. However, this application needs to be validated in a future study when the progression of sepsis would be assessed.

In a previous publication, the serum proteome of pigs with meningitis caused by *S. suis* was studied and they reported up to 316 differentially expressed proteins in the disease group [8]. The higher number of proteins observed could be due to the different methods used since they studied meningitis in an experimental-induced model of pigs. Interestingly, we found two proteins that matched with that encountered by this article: ALDOA and HRG. In our case, ALDOA was detected in saliva, being a possible useful marker for sepsis condition. HRG was decreased in the serum of our pigs with meningitis, but it was increased in experimentally induced meningitis [8]. Further studies would be necessary to elucidate the reason for the different behaviour of HRG in both studies. For example, a possible cause for this could be the different breeds used in both reports since the previous publication used Bama miniature pigs, while our study was made with Large White pigs.

### 3.3. Clinical Implications of Differentially Expressed Salivary Proteins in Pigs with Meningitis

The expression of different proteins in saliva could be related to the clinical alterations manifested by the pigs affected by meningitis. For instance, the increased expression of VCL is an indicator of muscle damage and seizures, while DSC2 showed more specificity for myocardial damage during *S. suis* infection [21]. Therefore, further studies could be performed to evaluate if the magnitude of the increase of these proteins could be associated with the presence of more severe clinical signs and also if the decrease of these proteins after treatment could predict improvement in the clinical sign of the pigs.

This study has some limitations that should be considered. First, the small population of pigs used in the proteomic study creates the need to confirm these findings in a larger population. Moreover, only male pigs were used in this study and sex differences have not been assessed in the proteomic study, so possible effects of gender should be considered. We preferred to only use animals of one sex in this report in order to avoid bias due to the influence of hormonal effects. In addition, future studies should address if haematology and serum biochemistry data could be correlated to proteomic findings. In this study, we investigated the presence of meningitis due to *S. suis,* but since meningitis can be multicausal (including non-septic causes), comparative studies using pigs with meningitis due to other septic and non-septic causes should be performed to contrast the relevance of proteins found in our study.

## 4. Materials and Methods

### 4.1. Animals

For the proteomic study, we used 20 male weaning pigs [(Sus scrofa domesticus) (Large White)], from a commercial farm located in the region of Murcia (Spain). The piglets were from 6 to 9 weeks old. They were divided into two groups: (1) the control group, consisting of clinically healthy pigs (HP, n = 10), and (2) the disease group, consisting of pigs diagnosed with meningitis due to *S. suis* (MP, n = 10). Allanimals in the disease group presented clinical symptomatology (ataxia, anorexia, lateral recumbency, and padding) [51] that raised suspicion of meningitis [32] and were positive to *S. suis* as described in point 4.3. No prior potential animal exclusion criteria were established.

For the validation of proteomic results, additional groups were included, consisting of 19 male pigs between 6 to 9 weeks old, diagnosed with *S. suis*-associated meningitis, and 19 healthy male pigs of the same age, sampled by the same approach that was used for the proteomic study.

All procedures were approved by the Ethical Committee on Animal Experimentation (CEEA) of the University of Murcia (protocol code CEEA 563/2019).

### 4.2. Saliva and Serum Collection

Paired saliva and serum were taken from pigs included in the study. Saliva was collected using a sponge clipped to a flexible thin metal rod approximately 20 cm in length. Pigs had thoroughly moistened the sponge by chewing, and then, the sponges were placed into Salivette^®^ tubes (Sarstedt, Aktiengesellschaft and Co. Nümbrecht, Germany).

After saliva collection, blood samples were obtained by puncturing the jugular vein and collected into vacuum plain tubes (BD Vacutainer, Franklin Lakes, NJ, USA).

All samples were kept at 4–8 °C in a portable refrigerator until arrival at the laboratory (in the next 15 min), where the vacutainer and the Salivette tubes were centrifuged at 3000× *g* and 4 °C for 10 min to obtain serum and saliva supernatant. Then, the aliquots were transferred into the Eppendorf tubes and stored at −80 °C until the analysis.

### 4.3. Streptococcus suis Isolation and Typification

Bacterial isolation and characterization were performed on the blood samples from clinically ill piglets. Samples were incubated on Columbia blood agar plates (Oxoid Ltd., Madrid, Spain) containing 5% defibrinated pig blood after 48 h at 37 °C under aerobic conditions, as previously reported [52]. The isolates were identified following standard procedures and confirmed by a PCR based on the glutamate dehydrogenase gene [53].

All animals of the meningitis group tested positive for *S. suis* serotype 9.

### 4.4. Proteomic Analysis

Proteomic analysis of saliva and serum samples was performed by a TMT-based quantitative approach as described previously [54].

Briefly, saliva samples were centrifuged (13,000× *g*, 10 min, 4 °C), and the proteins precipitated overnight by ice-cold acetone (VWR, Radnor, PA, USA). The pellet was resuspended in 1% SDS in 0.1 M triethyl ammonium bicarbonate (TEAB, Thermo Scientific, Rockford, IL, USA). The total protein concentrations in saliva and serum were measured with the commercial bicinchoninic acid-based reagent (Thermo Scientific, Rockford, IL, USA). The amount of 35 µg of the samples (saliva or serum) and internal standards (a pool of equal protein amount from all saliva or serum samples) were reduced using dithiothreitol (DTT) (Sigma-Aldrich, St. Louis, MO, USA), followed by alkylation with iodoacetamide (Sigma-Aldrich, St. Louis, MO, USA), and overnight precipitation with ice-cold acetone. The protein pellets were collected via centrifugation (9000× *g*, 4 °C, 15 min) and dissolved in 0.1 M TEAB prior to digestion with trypsin (Trypsin Gold, Promega; 1 mg/mL; trypsin-to-protein ratio 1:35, at 37 °C overnight). The next step was TMT (Thermo Scientific, Rockford, IL, USA) labelling, according to the manufacturer’s instructions. The 19 μL of the specific TMT label was mixed with each sample. Following the labelling for 60 min at room temperature, the reaction was stopped with 5% hydroxylamine (Sigma-Aldrich, St. Louis, MO, USA). A mixture of the five randomly chosen TMT-labeled samples and the internal standard was prepared for LC-MS/MS analysis.

The modular system integrating the Ultimate 3000 RSLCnano system (Dionex, Germering, Germany) and the Q Exactive Plus mass spectrometer (Thermo Fisher Scientific, Bremen, Germany) was used for LC-MS/MS analysis. Following their dissolution in the loading solvent (2% ACN, 0.1% formic acid in water), the labeled peptides were loaded onto the trap column (C18 PepMap100, 5 μm, 100A, 300 μm × 5 mm). After that, the separation on the analytical column (PepMap™ RSLC C18, 50 cm × 75 μm) followed. For achieving the separation gradient, we used two mobile phases, mobile phase A (0.1% formic acid in water) and mobile phase B (0.1% formic acid in 80% acetonitrile). The separation of the proteins was achieved by using the linear gradient of 5–55% mobile phase B over 120 min, followed by 55% to 95% for 1 min, 2 min at 95%, and decrease to 5% B during 20 min under the flow rate of 300 nL/min. The nanospray Flex ion source (Thermo Fisher Scientific, Bremen, Germany) with the 10 μm-inner diameters SilicaTip emitter (New Objective, Littleton, MA, USA) was used for ionization. The MS was operated as follows: positive ion mode using the DDA Top8 method, full scan in the range from m/z 350.0 to m/z 1800.0, resolution 70,000, 120 ms injection time, AGC target 1 × 10^6^, isolation window ± 2.0 Da, and the dynamic exclusion 30 s. The conditions for HCD fragmentation were stepped collision energy (29% and 35% NCE) with a resolution of 17,500 and an AGC target of 2 × 10^5^. The criteria to exclude the precursor ions from fragmentation were the unassigned charge state, or the charge states +1 and higher than +7 were excluded. The mass spectrometry proteomics data have been deposited to the ProteomeXchange Consortium via the PRIDE partner repository with the dataset identifier PXD037430.

For protein identification and quantification, we employed the SEQUEST algorithm with the Proteome Discoverer software (version 2.3., ThermoFisher Scientific, Waltham, MA, USA), searching against Sus scrofa FASTA files (downloaded from Uniprot database on 2 December 2020, 150,392 sequences). The identification parameters were: two trypsin missed cleavage sites, precursor, and fragment mass tolerances of 10 ppm and 0.02 Da, respectively; carbamidomethyl (C) fixed peptide modification, oxidation (M), and TMT six-plex (K, peptide N-terminus) dynamic modifications. The false discovery rate (FDR) was set at 5%, as calculated with the Percolator algorithm in the Proteome Discoverer workflow. Only the confidently identified proteins (at least two unique peptides and 5% FDR) entered the bioinformatics analysis.

### 4.5. Statistics and Bioinformatics Analyses

Statistical analyses of proteomics results were performed using R software v.4.1.2. [55], following a previously published protocol [56]. In brief, sample outliers were detected by Dixon’s test from R package outliers v0.14 and excluded from the further analysis, and the difference in protein abundance between MP and HP was accessed by the Mann–Whitney test. The *p*-values were adjusted using the false discovery rate (FDR) from R package qvalue v2.2.2, and differences with FDR < 0.05 were considered significant. Protein abundance fold changes between two groups were calculated as median (Group MP)/median (Group HP) and expressed on the log2 scale. Principal component analysis (PCA) and volcano plots were designed using the R package ggplot2 v3.1.1.

Further, the hierarchical clustering was analyzed for the proteins with the different relative abundance in saliva or serum between the piglets with meningitis and healthy piglets. The individual data were entered into the software (MetaboAnalyst™ version 5.0) without any transformation, and the clustering was based on the Euclidean distance.

The Protein Analysis Through Evolutionary Relationship (PANTHER) tool (http://www.pantherdb.org/, accessed on 20 July 2022) with the subset of GO terms (GO Slim database) was employed for functional enrichment analyses [57]. The REACTOME tool, using the human genome as the background, was accessed for the pathway enrichment analysis [58]. Significantly enriched pathways were considered for those with FDR-adjusted *p*-value < 0.05.

### 4.6. Validation Study

Among the proteins identified with the relative abundance in saliva differing between MP and HP, ADA was selected as a biomarker candidate for validation in an additional group of pigs with *S. suis*-associated meningitis (n = 19) which was compared with a group of healthy pigs (n = 19).

The activity of ADA was measured using an automated assay that was previously validated in the saliva of pigs [59]. Salivary ADA activity between pigs with meningitis and healthy pigs was compared using the Mann–Whitney U. Results were expressed as median and interquartile ranges, and the differences were considered significant when the *p*-value was below 0.05. Further, diagnostic accuracy was assessed via the Receiver Operating Characteristic (ROC) curve analysis, and the cut-off value, with optimal sensitivity and specificity, was determined. In the validation study, MedCalc™ software (version 16.2.1) was used for statistical analyses.

## 5. Conclusions

The analysis of the salivary and serum proteome of pigs with meningitis produced by *S. suis* evidence that this disease produced changes in proteins in both fluids. A similar number of proteins was changed in both saliva and serum, but their nature was different. VCL and DSC2 were the most upregulated proteins in saliva, both being related to muscle damage. The upregulation of ADA would reflect the immune system activation associated with sepsis. In serum, the proteins showed an upregulation of a serpin protein reflecting a protective response of the organism and an increase in HAPT indicating inflammation. Overall, the proteins changed to reflect the physiological mechanisms associated with meningitis in pigs caused by *S. suis* and could be potential biomarkers of this disease.

## Figures and Tables

**Table 1:** Pathway enrichment analysis of the saliva proteins differentially abundant between the piglets with meningitis and healthy piglets. FDR < 0.05 is considered significant.

Pathway	FDR	Count		Genes
		Observed	Background	
Platelet degranulation	<0.001	7	141	TF, ECM1, APOA1, A1BG, ALDOA, HRG, VCL
Response to elevated platelet cytosolic Ca2+	<0.001	7	148	TF, ECM1, APOA1, A1BG, ALDOA, HRG, VCL
Hemostasis	<0.001	10	803	IGHM, TF, ECM1, HBB, APOA1, A1BG, ALDOA, HRG, VCL
Platelet activation, signaling and aggregation	<0.001	7	293	TF, ECM1, APOA1, A1BG, ALDOA, HRG, VCL
Neutrophil degranulation	<0.001	8	480	TF, SERPINB12, GSN, KRT1, HBB, A1BG, ALDOA, VCL
Amyloid fiber formation	0.010	3	89	TF, GSN, APOA1
Innate immune system	0.026	8	1345	TF, SERPINB12, GSN, KRT1, HBB, A1BG, ALDOA, VCL
Formation of the cornified envelope	0.027	3	138	KRT1, DSG3, DSC2
Apoptotic cleavage of cellular proteins	0.029	2	38	GSN, DSG3

FDR—False discovery rate (FDR).

**Table 2:** Pathway enrichment analysis of the serum proteins differentially abundant between the piglets with meningitis and healthy piglets. FDR < 0.05 is considered significant.

Pathway	FDR	Count		Genes
		Observed	Background	
Platelet degranulation	6.305 × 10^5^	6	141	LOC100156325, TF, ALB, APOA1, HRG, CLU
Response to elevated platelet cytosolic Ca2+	1.859 × 10^4^	6	148	LOC100156325, TF, ALB, APOA1, HRG, CLU
Post-translational protein phosphorylation	0.002	4	109	TF, ITIH2, ALB, APOA1
Complement cascade	0.002	5	156	APCS, VTN, CLU, CPN2, CFB
Regulation of complement cascade	0.017	4	139	VTN, CLU, CPN2, CFB
Regulation of insulin-like growth factor (IGF) transport and uptake by insulin-like growth factor binding proteins (IGFBPs)	0.020	4	127	TF, ITIH2, ALB, APOA1
Neutrophil degranulation	0.020	5	480	LOC100156325, TF, MGAM, HP, LBP
Scavenging of heme from plasma	0.023	3	106	ALB, HP, APOA1
Platelet activation, signaling and aggregation	0.030	6	293	LOC100156325, TF, ALB, APOA1, HRG, CLU
Antimicrobial peptides	0.042	3	123	TF, LBP, CLU

## Data Availability

Proteomic data are available via ProteomeXchange with identifier PXD037430.

## Supplementary Materials

The following supporting information can be downloaded at: https://www.mdpi.com/article/10.3390/ijms232213700/s1.
