# Supplementary material for: Revealing the Changes in Saliva and Serum Proteins of Pigs with Meningitis Caused by Streptococcus Suis: A Proteomic Approach
Source: Int J Mol Sci. 2022 Nov 8;23(22):13700. doi: 10.3390/ijms232213700 (PMC9698074; doi:10.3390/ijms232213700)
Supplement: Supplementary file 1 [file ijms-23-13700-s001.zip › ijms-1969065-supplementary.pdf]

Supplementary data

Supplementary Table S1. Proteins with different relative abundances in the saliva of the piglets with meningitis (MP) and healthy piglets (HP).

Accession ID	Gene	Description	FC	Log2FC	P–value	FDR
Higher relative abundance in MP						
A0A4X1SRF3	VCL	Metavinculin	1.62	0.70	0.002	0.017
A0A287A191	DSC2	Desmocollin 2	1.58	0.66	0.004	0.023
A0A287ALC1	IGHM	Immunoglobulin heavy constant mu	1.57	0.65	0.003	0.018
A0A4X1U0N5	ALDOA	Fructose-bisphosphate aldolase	1.56	0.64	0.002	0.017
A0A4X1TUX8	SERPINB12	SERPIN domain-containing protein	1.53	0.61	0.003	0.018
A0A4X1SFT0	ECM1*	Extracellular matrix protein 1*	1.50	0.58	0.007	0.033
A0A4X1VL50	DSG3*	Desmoglein 3*	1.45	0.54	0.008	0.035
A0A4X1TTU1	ADA*	Adenosine aminohydrolase*	1.39	0.48	0.011	0.045
A0A287A6P1	GSN	Actin-depolymerizing factor	1.38	0.46	0.002	0.017
F1RFV3	VMO1	Vitelline membrane outer layer protein 1	1.30	0.38	0.006	0.030
Q4A3R3-2	DMBT1	Isoform 2 of Deleted in malignant brain tumors 1 protein	1.22	0.29	0.011	0.045
Lower abundance in MP						
A0A5G2R5N9	TF	Serotransferrin	0.86	-0.22	0.002	0.017
F1SGG3	KRT1	Cytokeratin-1	0.80	-0.32	0.001	0.013
P81245	OBP*	Odorant-binding protein*	0.79	-0.34	0.011	0.045
A0A5G2QYD6	HRG	Cystatin domain-containing protein	0.72	-0.47	0.002	0.017
A0A4X1TA63	CHIA	Chitinase	0.69	-0.54	0.001	0.013
K7GM40	APOA1	Apolipoprotein A-I	0.68	-0.56	0.001	0.013
A0A4X1W8Z7	A1BG*	Alpha-1B-glycoprotein*	0.63	-0.67	0.006	0.030
A0A287B057	OBP2B	Lipocln_cytosolic_FA-bd_dom domain-containing protein	0.59	-0.76	< 0.001	0.013
P02067	HBB	Hemoglobin subunit beta	0.55	-0.86	0.001	0.013
A0A4X1T2P9	LOC100739218	Double-headed protease inhibitor, submandibular gland-like	0.33	-1.60	< 0.001	0.013

FC – fold change, calculated as median (Group MP)/median (Group HP); FDR– false discovery rate; * after blasting in UniProt

Supplementary Table S2. Gene Ontology for the saliva proteins with the different abundance between piglets with meningitis and healthy piglets.

Ontology	Description	Genes	
		Number of genes	% of genes
Molecular Function	binding (GO:0005488)	11	40.7
	catalytic activity (GO:0003824)	7	25.9
	molecular function regulator (GO:0098772)	2	7.4
	structural molecule activity (GO:0005198)	1	3.7
Biological Process	biological adhesion (GO:0022610)	2	3.7
	biological process involved in interspecies interaction between organisms (GO:0044419)	3	11.1
	biological regulation (GO:0065007)	5	18.5
	biomineralization (GO:0110148)	1	3.7
	cellular process (GO:0009987)	11	40.7
	developmental process (GO:0032502)	3	11.1
	growth (GO:0040007)	1	3.7
	immune system process (GO:0002376)	3	11.1
	localization (GO:0051179)	4	14.8
	metabolic process (GO:0008152)	7	25.9
	multicellular organismal process (GO:0032501)	3	11.1
	biological adhesion (GO:0022610)	4	14.8
Cellular Component	cell junction (GO:0030054)	2	9.1
	cell periphery (GO:0071944)	6	27.3
	cytoplasm (GO:0005737)	10	45.5
	cytosol (GO:0005829)	4	18.2
	endomembrane system (GO:0012505)	4	18.2
	external encapsulating structure (GO:0030312)	1	4.5
	extracellular region (GO:0005576)	17	77.3
	extracellular space (GO:0005615)	16	72.7
	intracellular anatomical structure (GO:0005622)	11	50.0
	membrane (GO:0016020)	5	22.7
	organelle (GO:0043226)	7	31.8
	supramolecular complex (GO:0099080)	1	4.5
	protein-containing complex (GO:0032991)	2	7.4
Protein Class	cell adhesion molecule (PC00069)	2	7.4
	cytoskeletal protein (PC00085)	2	7.4
	defense/immunity protein (PC00090)	1	3.7
	metabolite interconversion enzyme (PC00262)	3	11.1
	protein-binding activity modulator (PC00095)	3	11.1
	transfer/carrier protein (PC00219)	10	37.0

Supplementary Table S3. The proteins with different relative abundances in the serum of the piglets with meningitis (MP) and healthy piglets (HP).

Accession ID	Gene	Description	FC	Log2FC	P–value	FDR
Higher relative abundance in MP						

A0A286ZYZ5	LOC106504547	SERPIN domain-containing protein	2.03	1.02	< 0.001	0.005
Q8SPS7	HP	Haptoglobin	1.81	0.85	< 0.001	< 0.001
O19063	APCS	Serum amyloid P-component	1.70	0.77	< 0.001	< 0.001
A0A4X1SH92	LOC396684*	SERPIN domain-containing protein	1.50	0.58	< 0.001	< 0.001
A0A4X1TJP8	LBP	Lipopolysaccharide-binding protein	1.45	0.54	0.002	0.015
F1SCC6	LOC100156325	SERPIN domain-containing protein	1.43	0.52	< 0.001	< 0.001
A0A4X1T8C9	CPN2*	Carboxypeptidase N subunit 2	1.23	0.30	0.010	0.047
Lower abundance in MP						
K7GPT9	CFB	C3/C5 convertase	0.92	-0.12	0.009	0.046
A2THZ2	ALB*	Albumin (Fragment)	0.86	-0.22	0.005	0.034
A0A480KXE8	MGAM*	Maltase-glucoamylase, intestinal	0.80	-0.33	0.002	0.015
A0A480QPG5	HGFAC*	Hepatocyte growth factor activator isoform 2 preproprotein	0.80	-0.31	0.001	0.012
A0A5S8KLN1	CLU	Clusterin	0.78	-0.36	0.007	0.040
A0A480SQD8	ITIH1*	Inter-alpha-trypsin inhibitor heavy chain H1 isoform	0.77	-0.39	< 0.001	< 0.001
A0A480RB65	TF*	Serotransferrin	0.77	-0.37	0.001	0.009
A0A4X1W718	AponN*	Beta-1 metal-binding globulin	0.77	-0.38	0.001	0.009
A0A4X1W7I8	ApoN*	Ovarian and testicular apolipoprotein N	0.75	-0.42	0.009	0.047
P48819	VTN	Vitronectin	0.74	-0.44	< 0.001	0.005
A0A5G2QEV5	ITIH2	Inter-alpha-trypsin inhibitor heavy chain H2	0.74	-0.43	< 0.001	0.005
K7GM40	APOA1	Apolipoprotein A-I	0.68	-0.57	0.007	0.040
A0A481B9A6	HRG*	Histidine-rich glycoprotein	0.58	-0.78	< 0.001	0.003

FC – fold change, calculated as median (Group MP)/median (Group HP); FDR– false discovery rate; * after blasting in UniProt

Supplementary Table S4. Gene Ontology for the serum proteins with the different abundance between piglets with meningitis and healthy piglets.

Ontology	Description	Genes	
		Number of genes	% of genes
Molecular Function	binding (GO:0005488)	8	27.6
	molecular function regulator (GO:0098772)	4	13.8
	catalytic activity (GO:0003824)	8	27.6
Biological Process	cellular process (GO:0009987)	9	31.0
	localization (GO:0051179)	6	20.7
	biological process involved in interspecies interaction between organisms (GO:0044419)	5	17.2
	biological regulation (GO:0065007)	8	27.6
	response to stimulus (GO:0050896)	9	31.0
	signaling (GO:0023052)	1	3.4
	developmental process (GO:0032502)	2	6.9
	multicellular organismal process (GO:0032501)	2	6.9

	biological adhesion (GO:0022610)	2	6.9
	metabolic process (GO:0008152)	6	20.7
	growth (GO:0040007)	2	6.9
	immune system process (GO:0002376)	5	17.2
Cellular Component	cell periphery (GO:0071944)	7	38.9
	membrane (GO:0016020)	4	22.2
	cytoplasm (GO:0005737)	6	33.3
	external encapsulating structure (GO:0030312)	3	16.7
	extracellular region (GO:0005576)	18	100.0
	intracellular anatomical structure (GO:0005622)	6	33.3
	endomembrane system (GO:0012505)	4	22.2
	organelle (GO:0043226)	4	22.2
	extracellular space (GO:0005615)	18	100.0
Protein Class	transmembrane signal receptor (PC00197)	1	3.4
	transfer/carrier protein (PC00219)	8	27.6
	defense/immunity protein (PC00090)	1	3.4
	protein-binding activity modulator (PC00095)	8	27.6
	metabolite interconversion enzyme (PC00262)	1	3.4
	protein modifying enzyme (PC00260)	3	10.3
